# Hot-Injection Synthesis of Cesium Lead Halide Perovskite Nanowires with Tunable Optical Properties

**DOI:** 10.3390/ma17102173

**Published:** 2024-05-07

**Authors:** Jiazhen He, Hang Li, Chengqi Liu, Xiaoqian Wang, Qi Zhang, Jinfeng Liu, Mingwei Wang, Yong Liu

**Affiliations:** State Key Laboratory of Advanced Technology for Materials Synthesis and Processing, International School of Materials Science and Engineering (ISMSE), Wuhan University of Technology (WUT), Wuhan 430070, China; jiazhenhe0606@163.com (J.H.); leehang@whut.edu.cn (H.L.); liuchengqi42@163.com (C.L.); 303568@whut.edu.cn (X.W.); zq13307239180@163.com (Q.Z.); liujinf990528@whut.edu.cn (J.L.); wmw1842591883@163.com (M.W.)

**Keywords:** perovskite nanowires, synthesis, photoelectric properties

## Abstract

Metal halide perovskite semiconductors have emerged as promising materials for various optoelectronic applications due to their unique crystal structure and outstanding properties. Among different forms, perovskite nanowires (NWs) offer distinct advantages, including a high aspect ratio, superior crystallinity, excellent light absorption, and carrier transport properties, as well as unique anisotropic luminescence properties. Understanding the formation mechanism and structure–property relationship of perovskite NWs is crucial for exploring their potential in optoelectronic devices. In this study, we successfully synthesized all-inorganic halide perovskite NWs with high aspect ratios and an orthorhombic crystal phase using the hot-injection method with controlled reaction conditions and surface ligands. These NWs exhibit excellent optical and electrical properties. Moreover, precise control over the halogen composition through a simple anion exchange process enables the tuning of the bandgap, leading to fluorescence emission, covering a wide range of colors across the visible spectrum. Consequently, these perovskite NWs hold great potential for efficient energy conversion and catalytic applications in photoelectrocatalysis.

## 1. Introduction

In recent years, metal halide perovskite semiconductors have garnered significant attention due to their rapid development in photovoltaic applications. Metal halide perovskite’s general formula for their structure is APbX_3_, where A is a monovalent cation with a large ionic radius, such as Cs^+^, MA^+^ (CH_3_NH_3_^+^), and FA^+^ (CH(NH_2_)_2_^+^); B is a divalent metal cation, such as Pb^2+^ and Sn^2+^; and X denotes halide ions, including Cl^−^, Br^−^, and I^−^ [1,2]. Based on the advantages of high absorption coefficients, adjustable bandgaps, wide spectral absorption ranges, high photovoltaic conversion efficiencies, high photoluminescent quantum yields (PLQY), and other advantages, metal halide perovskite can be widely applied in photovoltaic applications [3,4]. Because of their quantum yield and other advantages, metal halide perovskites have shown promising applications in optoelectronic devices such as photodetectors [5,6,7], solar cells [8,9,10], light-emitting diodes (LEDs) [11,12,13], and lasers [14,15,16]. Especially in the field of photovoltaics, over the past decade, the photoelectric conversion efficiency of metal halide perovskites has exhibited rapid development, with their photoelectric conversion efficiency escalating from an initial 3.8% to 26.14% [17], surpassing the mature alternatives, such as organic semiconductors and polycrystalline silicon. Compared with organic–inorganic hybridized perovskites, all-inorganic perovskites have higher stability and excellent optical properties [18], showing great potential for photoelectrocatalytic applications.

Different morphologies of perovskite materials, including bulk crystals [19,20], thin films [21,22], nanocrystals (NCs) [23,24], two-dimensional nanosheets [25,26], one-dimensional nanowires (NWs) [27,28], and zero-dimensional quantum dots (QDs) [29], can be obtained by different synthesis processes. Among them, researchers have gained interest in one-dimensional perovskite NWs with high aspect ratios, unique carrier transport channels, and distinctive anisotropic luminescence properties [30]. Compared with other morphologies, perovskite NWs provide guided channels for the transport and the propagation of carriers and photons along one dimension while constraining their movement in the other two dimensions [31], the unique property that has potential applications in next-generation high-performance optoelectronic devices.

Currently, the method of synthesizing perovskite nanocrystals via hot injection has reached a high level of maturity [32]. Vighnesh et al. [33] summarized a thermal injection synthesis scheme for preparing high-quality CsPbBr_3_ nanocrystals and outlined both intrinsic and extrinsic factors that influence their reproducibility. Most of the perovskite nanowire synthesis processes are based on the synthesis experience of perovskite nanocrystals and other semiconductor NWs. Wang et al. [34] demonstrated a colloidal synthesis method for directly synthesizing stable single-crystal formamidinium (FA) cesium double-cation bromide lead FA_0_._33_Cs_0_._67_PbBr_3-x_I_x_ (0 ≤ x ≤ 3) perovskite nanostructures by adjusting the reaction ligands and halide compositions, yielding nanowires and nanoplates. Zhang et al. [35] optimized reaction parameters to synthesize cubic-phase CsPbBr_3_ nanowires with an aspect ratio of approximately 19 during the synthesis of CsPbBr_3_ nanocrystals. However, the synthesis of perovskite NWs has yet to be investigated in detail. In synthesizing most semiconductor NWs, reaction temperature, reaction time, and surface ligands play an important role. It has been shown that a lower reaction temperature is favorable to make the semiconductor NWs grow anisotropically [36], reaction time affects the evolution of semiconductor morphology [37], and surface ligands not only passivate surface defects but also have the potential to alter the reaction kinetics of semiconductor materials [38]. Therefore, the reaction temperature, reaction time, and surface ligands significantly impact the growth and optical properties of perovskite NWs.

Herein, CsPbBr_3_ perovskite NWs characterized by a high aspect ratio and an orthorhombic crystal phase were successfully synthesized using the hot-injection method, with tuned the reaction time, reaction temperature, and surface ligands. The morphology, structure, and composition of the CsPbBr_3_ NWs were characterized. The results show that the CsPbBr_3_ nanowire has a diameter of about 10 nm and a length extending several tens of micrometers. It has a well-defined orthorhombic phase structure and predominantly grows along the [001] crystallographic direction. The surface of the CsPbBr_3_ NWs is covered by an organic amine ligand, and the presence of oleic acid, originating from cesium oleate, was detected. The optical properties of CsPbBr_3_ NWs and the electrochemical impedance and photocurrent density in sodium sulfate solution were also evaluated. The results showed that the CsPbBr_3_ NWs were prone to anion exchange reactions, and the exchanged NWs had a bright and tunable PL covering almost the entire visible spectral region. Compared to CsPbBr_3_ NCs, the CsPbBr_3_ NWs had minor electrochemical impedance and a higher photocurrent density, suggesting their potential photoelectrocatalytic applications.

## 2. Materials and Methods

### 2.1. Materials

Cesium carbonate (Cs_2_CO_3_, 99.9%, Aladdin, Shanghai, China), oleic acid (OA, 80–90%, Aladdin), 1-octadecene (ODE, >90% (GC), Aladdin), lead (II) bromide (PbBr_2_, 99.0%, Aladdin), oleylamine (OAm, 80–90%, Aladdin), dodecylamine (98%, Aladdin), octylamine (99%, Aladdin), hexylamine (99%, Aladdin), butylamine (≥99% (GC), Aladdin), lead (II) chloride (99.99% metals basis, Aladdin), lead (II) iodide (99.99% metals basis, Aladdin), trinoctylphosphine (TOP, 90%, Aladdin), cyclohexane (AR, 99.5%, Aladdin), and toluene were purchased from Sinopharm Chemical Reagent Co., Ltd., Shanghai, China and organic amine ligands were dried with molecular sieves.

### 2.2. Methods

#### 2.2.1. Preparation of CsOA Precursors

A typical synthesis method is to add 400 mg (1.23 mmol) of Cs_2_CO_3_, 15 mL of ODE, and 1.4 mL of OA into a 50 mL three-necked flask. A double-row tube was connected and dried under vacuum at 120 °C for 1 h. Then, the temperature was raised to 150 °C until the powder was completely dissolved and the solution became clear. The synthesized cesium oleate solidifies at room temperature, so it needs to be preheated to 120 °C before synthesizing CsPbBr_3_ NWs.

#### 2.2.2. Synthesis of CsPbBr_3_ NWs

Briefly, 0.0734 mg (0.2 mmol) of PbBr_2_ and 5 mL of ODE were added to another 50 mL three-neck flask and dried under vacuum at 120 °C for 30 min. Then, 6.8 mmol of organic amine ligand (butylamine, hexylamine, octylamine, dodecylamine, oleylamine, or a mixture of the two organic amines) was slowly injected under the N_2_ atmosphere. Subsequently, the temperature was raised to a specific temperature (105 °C, 125 °C, 145 °C, 165 °C, and 185 °C) and kept for 10 min to stabilize the mixture, which was milky white. Then, 0.8 mL of cesium oleate was rapidly injected. The reaction was performed for a specific time (5 s, 1 min, 5 min, 10 min, 40 min, and 60 min), and the solution turned yellow (the longer the reaction time, the darker the color). The reaction was quenched in an ice-water bath. The sample was purified by centrifugation and subsequently dispersed in cyclohexane for further use.

#### 2.2.3. Preparation of PbX_2_ (X = Cl, I) Solution

We put 0.4 mmol of PbX_2_ (X = Cl, I), 0.5 mL of OA, 0.5 mL of OAm, 2 mL of TOP, and 7 mL of toluene into a 25 mL glass vial and stirred the mixture in an oil bath at 100 °C for 2 h until the PbX_2_ powder is completely dissolved. We then cooled it to room temperature and set it aside. The PbCl_2_ stock is a white transparent liquid, and the PbI_2_ stock solution is a yellow transparent liquid.

#### 2.2.4. Anion Exchange Reactions

Briefly, the CsPbBr_3_ nanowire solution was diluted 10 times, and different volumes of PbX_2_ stock solution were added to the dilute solution to initiate the anion exchange reaction. After the reaction was completed, centrifugation was performed at 5000 rpm for 8 min, and the nanowire precipitates were taken and dispersed in cyclohexane for backup. CsPbBr_x_Cl_3−x_ (0 < x < 3) and CsPbBr_y_I_3−y_ (0 < y < 3) NWs were obtained, respectively.

### 2.3. Characterization

#### 2.3.1. Characterization of Material Morphology

OM images were obtained using an RX50M series metallographic microscope (Sunny Optical Technology (Group) Company Limited, Yuyao, China) equipped with Lightool software (x64,4.8.15957.20191115). SEM images were obtained by Hitachi S-4800 field emission scanning microscope (5 KV) from Tokyo, Japan. The surface of the samples was sprayed with gold. TEM images were obtained using a 120 KV JEOL JEM-1400Plus transmission electron microscope (Beijing, China).

#### 2.3.2. Characterization of Material Structure

HRTEM images were obtained on a Talos F200S-type field emission transmission electron microscope (Hillsboro, OR, USA) at 200 KV. XRD images (λ = 1.540 Å, 40 kV, 40 mA, angular reproducibility 0.0001°) were recorded on a D8-ADVANCEX type X-ray diffractometer (Bruker, Karlsruhe, Germany), using a Cu target, and the range of the diffraction angle (2θ) was 10–60°, and the test samples were prepared by dropping a concentrated solution of the NWs on a clean silicon substrate. The Fourier-transform infrared (FTIR) spectra of the samples were recorded using a Nicolet 6700 FTIR spectrometer (Waltham, USA), and the test samples were prepared by dropping a concentrated solution of the NWs on a clean glass sheet. The full and elemental high-resolution spectra of the XPS were obtained using an AXIS SUPRA X-ray photoelectron spectrometer (Kratos, Manchester, UK), and the data were processed using Avantage (5.99), combined with an internal reference of the energy scale C1s peak (binding energy of C–C = 284.8 eV).

#### 2.3.3. Optical Characterization of Materials

The NWs underwent analysis via UV-Vis absorption spectroscopy utilizing a Shimadzu UV-1800 spectrophotometer (Shanghai, China) coupled with UV probe 2.52 software. Fluorescence spectra of the NWs were analyzed using a Shimadzu RF6000 spectrophotometer (Kyoto, Japan) equipped with LabSolutions RF software (1.11) with an excitation wavelength of 360 nm. The time-resolved photoluminescence spectra (TRPL) of the CsPbBr_3_ NWs were collected by a TCSPC-correlated single photon counter, using a fluorescence steady-state transient test system (PTI QuantaMaster^TM^ 4CW, Horiba, Austin, TX, USA). The test samples were prepared by mounting dilute solutions of the NWs in a four-way quartz cuvette.

#### 2.3.4. Electrochemical Characterization of Materials

Photocurrent and electrochemical impedance profiles of CsPbBr_3_ NWs were obtained using a Zennium electrochemical analyzer from Zahner Company (Kronach, Germany). The measurements were performed in a three-electrode setup with the sample electrode as the working electrode, a platinum sheet as the counter electrode and Ag/AgCl (saturated KCl) as the reference electrode (Ag/AgCl = +0.1989 V vs. NHE) in an electrochemical electrolyte (0.5 M Na_2_SO_4_). For photocurrent measurements, the light source was a 405 nm LED, and light-intensity tests were performed using a Newport photometer (Irvine, CA, USA). The electrochemical impedance spectra of 0.5 M Na_2_SO_4_ with an amplitude of 10 mV (frequency: 100 mHz~20 kHz) were measured at −0.1 V vs. NHE [39].

## 3. Results

**Figure 1:**
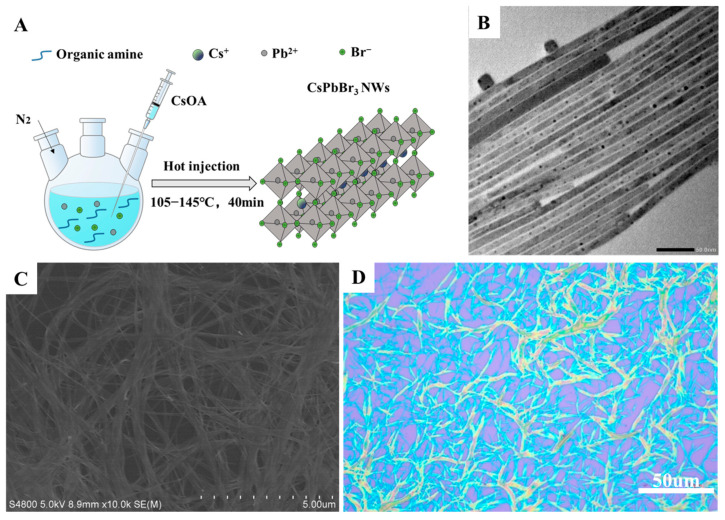
(**A**) Schematic diagram of the synthesis procedure, (**B**) TEM image, (**C**) SEM image, and (**D**) optical image of all-inorganic CsPbBr_3_ perovskite nanowires (NWs).

First, as shown in Figure 1A, cesium oleate (CsOA) obtained by the reaction of cesium carbonate with oleic acid was used as the precursor of cesium. Lead bromide served as lead and bromine sources, with organic amines acting as surface ligands and octadecene as the solvent. The CsPbBr_3_ nanowires (NWs) were synthesized by reacting these components at a specific temperature for a predetermined duration, and the detailed experimental procedure is described in the Section 2. In order to investigate the formation process of CsPbBr_3_ NWs, we prepared samples with different reaction times. Supplementary Figures S1 and S2 show that the samples formed a high aspect ratios thread within 5 s. However, the XRD pattern indicated (Supplementary Figure S3) that the thread was not a perovskite structure but an intermediate product. Subsequently, the intermediate product thread fractured and recrystallized into 80–100 nm CsPbBr_3_ perovskite nanorods (NRs), aggregating around the remaining thread. With time, the CsPbBr_3_ NRs gradually lengthened into CsPbBr_3_ NWs, and the intermediate products disappeared. At 40 min, phase-pure CsPbBr_3_ perovskite NWs can be obtained. Extending the reaction time to 60 min led to the aggregation of nanowires into bundles with poor dispersion and the formation of a by-product, Cs_4_PbBr_6_. Therefore, the optimal reaction time was determined to be 40 min.

The reaction temperature has a significant influence on the nucleation and growth of nanomaterials [40]. Supplementary Figures S4 and S5 show that CsPbBr_3_ NWs can be obtained at lower temperatures. However, with an increase in temperature, the dispersity of the CsPbBr_3_ NWs deteriorates, and some by-products begin to appear. At 145 °C, a considerable number of cubic by-products emerges. At 165 °C, larger aggregates approximately 500 nm in size are observed. At 185 °C, CsPbBr_3_ NWs have almost disappeared, and only several amorphous bars can be observed. Therefore, high-quality CsPbBr_3_ NWs can be synthesized at lower temperatures, at least below 145 °C.

According to previous reports, the surface ligands of CsPbBr_3_ nanocrystals are oleic acid and oleylamine [3], while in the synthesis of the previously reported perovskite NWs, only oleylamine and octylamine were added, in addition to a small amount of oleic acid introduced in CsOA [41]. Therefore, the organic amine ligands significantly impact the synthesis of CsPbBr_3_ NWs. In order to understand their role, experiments were conducted using a single type of organic amine ligand. In addition to oleylamine and octylamine, organic amines with different chain lengths, such as butylamine, hexylamine, and dodecylamine, were selected. In subsequent sections of the article, butylamine, hexylamine, octylamine, dodecylamine, and oleylamine are denoted as C4, C6, C8, C12, and C18, respectively, according to the difference in carbon chain length. As shown in Supplementary Figures S6 and S7, when short-chain organic amines were used, nanowires could not be grown, or the grown nanowires had unclear boundaries. With medium-length organic amines, the growth of nanowires was observed with clear boundaries, and due to the high aspect ratios, the nanowires tended to form bundles, which could be observed under an optical microscope. Moreover, nanowire bundles synthesized with longer organic amines were longer, indicating better dispersion and length of the nanowires. When oleylamine was used, shorter nanowires with better dispersion were observed. The growth of nanowires was also observed when using a mixed organic amine ligand (C8 and C18), with diameters of about 10 nm and lengths reaching several tens of micrometers. Therefore, medium-length organic amine ligands or mixed organic amine ligands can facilitate the growth of nanowires with high aspect ratios.

**Figure 2:**
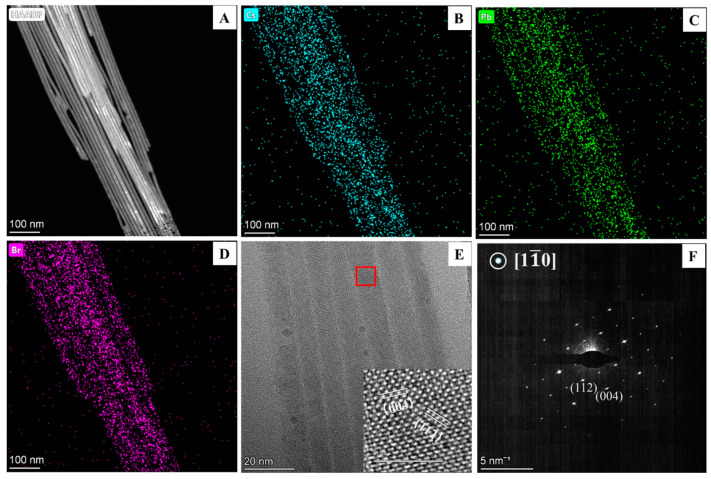
(**A**) HAADF-STEM; (**B**–**D**) STEM-EDS mapping of Cs, Pb, and Br; (**E**) HRTEM image; and (**F**) SADE image of the CsPbBr_3_ NWs. Inset of (**E**) is a localized enlarged HRTEM image of the red box.

For further discussion, we consider the synthesis conditions with a reaction time of 40 min and a reaction temperature of 125 °C, using C8 and C18 as surface ligands. Figure 1B–D show that the synthesized CsPbBr_3_ NWs have an average diameter of approximately 10 nm and can reach lengths of several tens of micrometers. The significant aspect ratio of these CsPbBr_3_ NWs facilitates their aggregation and entanglement under van der Waals forces, leading to a bundled arrangement observable under SEM and OM [42]. The elemental distribution of the CsPbBr_3_ NWs was examined through an Energy Dispersive Spectrometer (EDS) attached to the TEM, revealing a relatively uniform distribution of Cs, Pb, and Br elements within the CsPbBr_3_ NWs, with an atomic ratio close to Cs:Pb:Br ≈ 1:1:3 (Figure 2A–D). HRTEM images analyzed the crystal structure of the CsPbBr_3_ NWs. Clear lattice fringes of CsPbBr_3_ NWs are visible in Figure 2E, indicating high crystallinity. A magnified view of the lattice fringes on a single nanowire, as in the inset of Figure 2E, and the spacing of the lattice stripes in two directions is 0.29 nm (perpendicular to the growth direction) and 0.41 nm, respectively, which matches with the (004) and (112) faces of the orthorhombic phase CsPbBr_3_, suggesting that the CsPbBr_3_ NWs grows along the [001] direction. Compared to the nanocrystals, the excess organic amine ligand favors the growth of CsPbBr_3_ NWs along the [001] direction. The selected zone electron diffraction corresponding to Figure 2E is shown in Figure 2F. The crystallographic band axis is [1$\bar{1}$0], with the main diffraction spots matching well with the HRTEM results.

**Figure 3:**
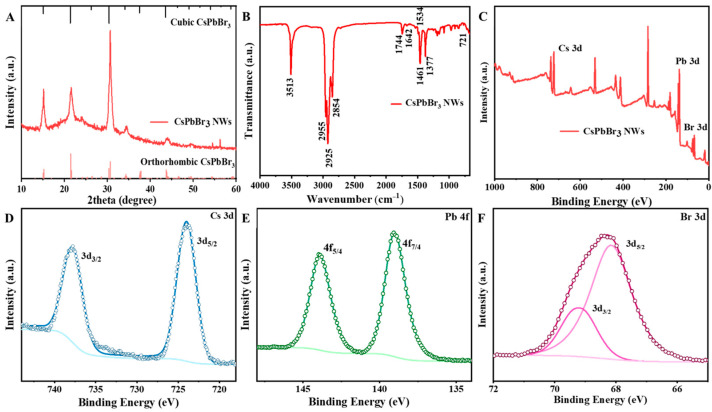
(**A**) XRD pattern, (**B**) FTIR spectra, (**C**) full XPS spectra, and (**D**–**F**) XPS high-resolution spectra of Cs3d, Pb4f, and Br3d of the CsPbBr_3_ NWs.

The structure of CsPbBr_3_ NWs was further analyzed using XRD. CsPbBr_3_ NWs have cubic, monoclinic, and orthorhombic phase structures, and CsPbBr_3_ nanocrystals are generally reported to have cubic phase structures [43]. Since the differences in the diffraction peaks of XRD for several structures are small, the physical phase of CsPbBr_3_ NWs needs to be determined more carefully. As shown in Figure 3A, the upper axis is the standard XRD pattern of the cubic phase (PDF#00-054-0752), and the lower axis is the XRD pattern of the orthorhombic phase (PDF#01-072-7929). It can be seen that the main difference between the orthorhombic phase and the cubic phase is the double peaks located at 15°, 21°, and 30°. A broader diffraction peak can be observed in the figure around 15°, 21°, and 30° each, but it is not simply categorized as a cubic phase. Therefore, in order to get a clearer picture of the physical phase of the CsPbBr_3_ perovskite NWs, we locally zoomed in on the peaks at 15°, 21°, and 30° (Supplementary Figure S8) and found that all three peaks had a weak small peak. A clear double peak could not be seen in the full spectrum at 10–60°, probably due to the wider half-peak widths and localized peak intensities of the CsPbBr_3_ NWs. Thus, the double peaks at 15°, 21°, and 30° confirm that the CsPbBr_3_ NWs were grown in the orthorhombic phase. Compared with the PDF cards of the orthorhombic phase, the diffraction peaks located at 15.05° and 15.2° corresponded to the (002) and (110) crystal planes, respectively; the diffraction peaks located at 21.4° and 21.6° corresponded to (020) and (200) crystal planes, respectively; and the diffraction peaks located at 30.4° and 30.69° correspond to the (004) and (220) crystal planes, respectively. The diffraction peak corresponding to the (004) crystal plane is relatively sharp, which further demonstrates the optimal growth of CsPbBr_3_ NWs along the [001] direction.

In order to investigate the functional groups on the surface of the samples, Fourier-transform infrared (FTIR) spectroscopy of the CsPbBr_3_ NWs was carried out. The characteristic peaks at 721, 2854, and 2925 cm^−1^ corresponded to the in-plane swaying vibrations and symmetric and asymmetric stretching vibrations of –CH_2_–. The characteristic peaks at 1377, 1461, and 2955 cm^−1^ are derived from symmetric and asymmetric bending vibration and antisymmetric stretching vibration of –CH_3_ [44]. Meanwhile, the characteristic peak at 1642 and 1744 cm^−1^ corresponds to the asymmetric and symmetric stretching vibrations of carboxylate –COO^−^ groups, indicating that the oleic acid introduced by CsOA exists on the sample surface in the form of carboxylate. The peaks at 1534 cm^−1^ correspond to the shear deformation vibration of –N–H of –NH_2_, and the strong characteristic peak of 3513 cm^−1^ corresponds to the N-H asymmetric stretching vibrations, providing evidence for the presence of oleamine molecules in the sample.

In addition, the surface chemical valence states of CsPbBr_3_ NWs were analyzed using XPS. The full spectrum of CsPbBr_3_ NWs and the binding energy profiles of Cs 3d, Pb 4f, and Br 3d were recorded in Figure 3B–F. The peaks of Cs 3d_5/2_ and Cs 3d_3/2_ were located near 724 eV and 738 eV, respectively, and the peaks of Pb 4f_7/4_ and Pb 4f_5/4_ were located near 139 eV and 144 eV neighborhoods, corresponding to Cs^+^ and Pb^2+^. The coupled peaks near 68 eV and 69 eV are from Br 3d_5/2_ and Br 3d_3/2_, corresponding to Br^−^. This analysis suggests that the binding energies for Cs, Pb, and Br in CsPbBr_3_ NWs are consistent with previous findings, lying within the margin of error [45]. Full XPS spectra show the elemental composition ratio of the CsPbBr_3_ NWs with an elemental content ratio of Cs:Pb:Br ≈ 1:1.4:2.95.

**Figure 4:**
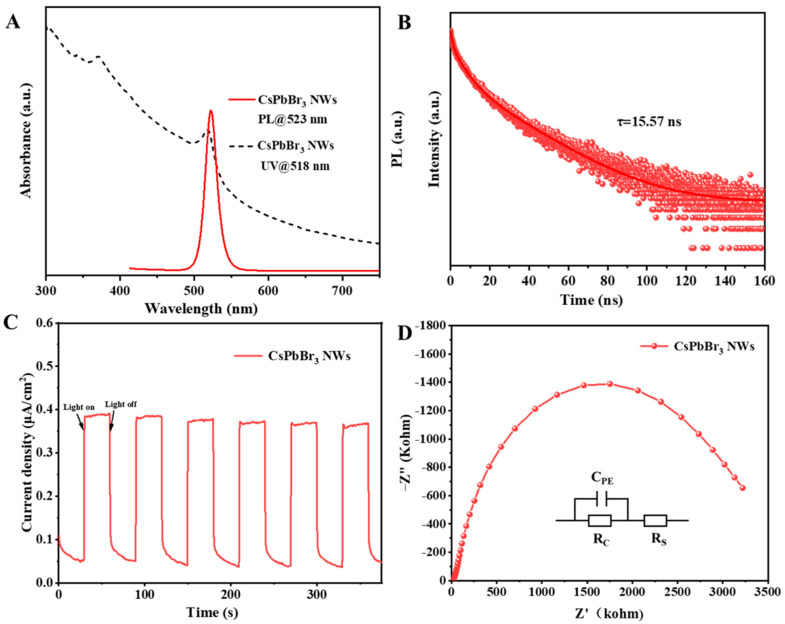
(**A**) UV-Vis absorption and photoluminescence (PL) spectra. (**B**) Time-resolved photoluminescence (TRPL) spectra of the CsPbBr_3_ NWs. (**C**) Transient photocurrent responses to on–off illumination of the CsPbBr_3_ NWs electrodes at −0.1 V versus NHE in neutral water (0.5 M Na_2_SO_4_). (**D**) Electrochemical impedance spectra (Nyquist plot) of the CsPbBr_3_ NWs.

Subsequently, the optical properties of the CsPbBr_3_ NWs were characterized by UV-Vis absorption spectra, fluorescence luminescence spectra, and fluorescence lifetime, as shown in Figure 4A,B. The absorption peak of CsPbBr_3_ NWs is 518 nm, and the fluorescence luminescence peak is 523 nm, which is in the green luminescence band. The fluorescence luminescence peak is red-shifted by 5 nm compared to the absorption peak, which the Stokes shift can explain. This redshift phenomenon indicates that CsPbBr_3_ nanowires can effectively convert absorbed light energy into luminescent energy, thus enhancing their photocatalytic performance. While the luminescence peak of the bulk perovskite single crystal is located around 530 nm, the blue shift of the nanowire luminescence peak is due to the diameter of the nanowire being around 8–10 nm, which is close to the Bohr radius (7 nm) of the CsPbBr_3_ perovskite, the quantum limited-domain effect leads to the change in the nanowire blue shift [46]. The time-resolved photoluminescence (TRPL) spectra of CsPbBr_3_ NWs (Figure 4B) were calculated through a three-exponential fitting. The average lifetime of CsPbBr_3_ NWs was determined to be approximately 15.57 ns. The longer fluorescence lifetime of CsPbBr_3_ nanowires is attributed to the longer carrier diffusion length in highly crystalline CsPbBr_3_ nanowires, helping improve the utilization of photo-generated carriers and enhance photocatalytic performance.

Then, the electrochemical impedance spectra and photocurrent responses of CsPbBr_3_ NWs were tested, as shown in Figure 4C,D. The Nyquist plots of the EIS spectra of the CsPbBr_3_ NWs showed a semicircular shape, and the corresponding equivalent circuit model was shown in the inset, where Cpe represents the double-layer capacitance, Rs represents the solution resistance between the reference electrode and the working electrode, and Rc signifies the charge-transfer resistance at the electrode interface. The fitting values of EIS are shown in Supplementary Table S1. Compared to the reported CsPbBr_3_ NCs that we studied before [46], the semicircular arcs of CsPbBr_3_ NWs are minor, indicating that the charge-transfer resistance of CsPbBr_3_ NWs is minor. Figure 4D shows the transient photocurrent response of CsPbBr_3_ NWs in 0.5 M Na_2_SO_4_ aqueous solution at −0.1 V vs. NHE. Under 405 nm light irradiation, the photocurrent density of CsPbBr_3_ NWs was about 0.38, which was also slightly higher than that of the reported CsPbBr_3_ NCs. Therefore, the minor charge-transfer resistance and larger photocurrent proved that the CsPbBr_3_ NWs had better carrier mobility efficiency and charge-transport properties, and the CsPbBr_3_ NWs had a broader prospect for application in photoelectrocatalysis.

**Figure 5:**
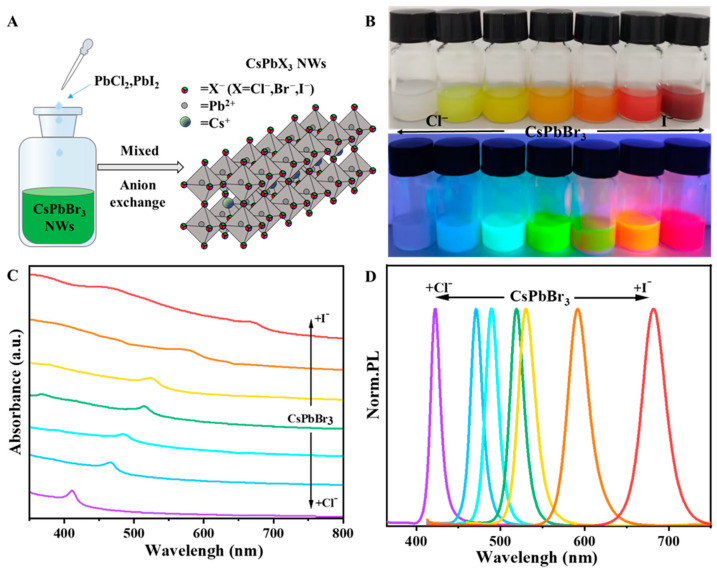
(**A**) Schematic diagram of the anion exchange reactions all-inorganic CsPbX_3_ (X = Cl, Br, I) NWs. (**B**) Images of CsPbX_3_ (X = Cl, Br, I) NWs solutions under sunlight and 365 nm UV lamp, respectively. (**C**) UV-Vis absorption spectra and (**D**) photoluminescence (PL) spectra of CsPbX_3_ (X = Cl, Br, and I) NWs.

Owing to the pronounced mobility exhibited by halide ions and the inherent rigidity of the cationic sublattice within halide perovskites, facile anion-exchange reactions ensue with notable expediency [47,48]. Perovskite NWs with different halide compositions can be obtained by mixing diluted CsPbBr_3_ NWs with different volumes of PbI_2_ or PbCl_2_ stock solutions, as shown in Figure 5A. Figure 5B shows that, under natural light, with the addition of Cl^−^ ions, the nanowire solution gradually changed from yellow to light and finally faded to white; with the addition of I^−^ ions, the nanowire solution gradually changed from yellow to orange, then to red, and finally to brown. Under the irradiation of a UV lamp, with the addition of Cl^−^ ions, the nanowire solution changed from green light to indigo light to blue light and finally showed a weak violet light; with the addition of I^−^ ions, the nanowire solution changed from blue light to a weak yellow light to orange light and finally showed a bright red light. After anion exchange, the addition of Cl^−^ and I^−^ does not destroy the morphology of the NWs, and Cs, Pb, Br, and Cl (I) are uniformly distributed in the NWs. The content of Br is significantly reduced, and the HRTEM images show the single-crystalline nature of the exchanged CsPbBr_x_Cl_3−x_ (0 < x < 3) and CsPbBr_y_I_3−y_ (0 < y < 3) NWs and the absence of epitaxial interfaces and grain boundaries (Supplementary Figures S8 and S9). The optical properties of CsPbBr_x_Cl_3−x_ (0 < x < 3) and CsPbBr_y_I_3−y_ (0 < y < 3) NWs were characterized by UV-Vis absorption and photoluminescence spectra (Figure 5C,D). The luminescence of CsPbBr_3_ perovskite NWs had the peak luminescence peaks at 523 nm, which were gradually red-shifted to 530 nm, 592 nm, and finally 682 nm with the addition of I^−^. With the addition of Cl^−^, the peak was gradually blue-shifted to 489 nm. With the addition of Cl, the peaks gradually shifted to 489 nm, 471 nm, and finally to 423 nm, the shortest wavelength. At the same time, the UV–visible absorption peaks were also blue-shifted and red-shifted, and the absorption peaks were located at 411 nm, 466 nm, 482 nm, 518 nm, 522 nm, 577 nm, and 677 nm, respectively. The graphs only represent the luminescence wavelengths of the seven samples in the experiment, and the amounts of PbI_2_ and PbCl_2_ reserve solution can also be adjusted to obtain NWs with different luminescence wavelengths. Thus, by adjusting the amount of added Cl^−^ and I^−^ ions, we can obtain CsPbX_3_ alloy NWs with various compositions while retaining favorable orthorhombic phases. The NWs with bright and tunable PL, covering almost the entire visible spectral region, can achieve more efficient energy conversion and catalytic reactions, providing new possibilities in the field of photoelectrocatalysis.

## 4. Conclusions

In summary, CsPbBr_3_ perovskite NWs characterized by a high aspect ratio and an orthorhombic crystal phase were successfully synthesized using the hot-injection method. A longer reaction time (40 min), a lower reaction temperature (below 145 °C), and medium chain-length organic amine ligands or mixed organic amine ligands are more favorable for the growth of CsPbBr_3_ NWs. A series of characterizations (HRTEM, XRD, FTIR, and XPS) confirmed the high-quality morphology, structure, and composition of the CsPbBr_3_ NWs. The CsPbBr_3_ NWs have excellent optical properties with bright green fluorescence emission at 523 nm, and the average PL lifetime is approximately 15.57 ns. Moreover, simple anion exchange processes enable the modulation of the NW composition, thereby tuning the bandgap and achieving fluorescent emission across almost the entire visible spectrum. Compared to previously reported nanocrystals, CsPbBr_3_ NWs exhibit lower electrochemical impedance and a higher corresponding photocurrent density. This indicates that perovskite nanowires possess distinct advantages and potential in the field of photoelectrocatalysis.

## Data Availability

Data are contained within the article and Supplementary Materials.

## Supplementary Materials

The following supporting information can be downloaded at https://www.mdpi.com/article/10.3390/ma17102173/s1, Figure S1. OM images of CsPbBr_3_ samples prepared with different reaction times. Scale bar, 10 μm; Figure S2. TEM images of CsPbBr_3_ samples prepared with different reaction times. Scale bar, 100 nm; Figure S3. XRD spectra of CsPbBr_3_ samples prepared with different reaction times; Figure S4. OM images of CsPbBr_3_ samples prepared with different reaction temperatures. Scale bar, 10 μm; Figure S5. TEM images of CsPbBr_3_ samples prepared with different reaction temperatures. Scale bar, 100 nm; Figure S6. OM images of CsPbBr_3_ samples prepared with organic amine ligands with different chain lengths. Scale bar, 10 μm; Figure S7. TEM images of CsPbBr_3_ samples prepared with organic amine ligands with different chain lengths. Scale bar, 100 nm; Figure S8. The locally amplified XRD spectra of the CsPbBr_3_ NWs at (**A**) 12–18°, (**B**) 19–24°, and (**C**) 28–33°; Table S1. EIS fitting parameters of CsPbBr_3_ NWs; Figure S9. (**A**) HAADF-STEM and (**B**–**E**) STEM-EDS mapping of Cs, Pb, Cl, and Br of the CsPbBr_x_Cl_3−x_ (0 < x < 3) NWs; (**F**) HRTEM image of the CsPbBr_x_Cl_3−x_ (0 < x < 3) NWs; Figure S10. (**A**) HAADF-STEM and (**B**–**E**) STEM-EDS mapping of Cs, Pb, I, and Br of the CsPbBr_y_I_3−y_ (0 < y < 3) NWs; (**F**) HRTEM image of the CsPbBr_y_I_3−y_ (0 < y < 3) NWs.
