# Supplementary material for: Hot-Injection Synthesis of Cesium Lead Halide Perovskite Nanowires with Tunable Optical Properties
Source: Materials (Basel). 2024 May 7;17(10):2173. doi: 10.3390/ma17102173 (PMC11123179; doi:10.3390/ma17102173)
Supplement: Supplementary file 1 [file materials-17-02173-s001.zip › materials-2936372-supplementary.pdf]

Supporting Information

Hot-Injection Synthesis of Cesium Lead Halide Perovskite Nanowires with Tunable Optical Properties

Jiazhen He, Hang Li, Chengqi Liu, Xiaoqian Wang, Qi Zhang, Jinfeng Liu, Mingwei Wang, and Yong Liu *

State Key Laboratory of Advanced Technology for Materials Synthesis and Processing, International School of Materials Science and Engineering (ISMSE), Wuhan University of Technology, Wuhan 430070, China; jiazhenhe0606@163.com (J.H.); leehang@whut.edu.cn(H.L.); liuchengqi42@163.com(C.L.); 303568@whut.edu.cn (X.W.); zq13307239180@163.com(Q.Z.); wmw1842591883@163.com(M.W.); liujinf990528@whut.edu.cn (J.L)

* Correspondence: liuyong3873@whut.edu.cn

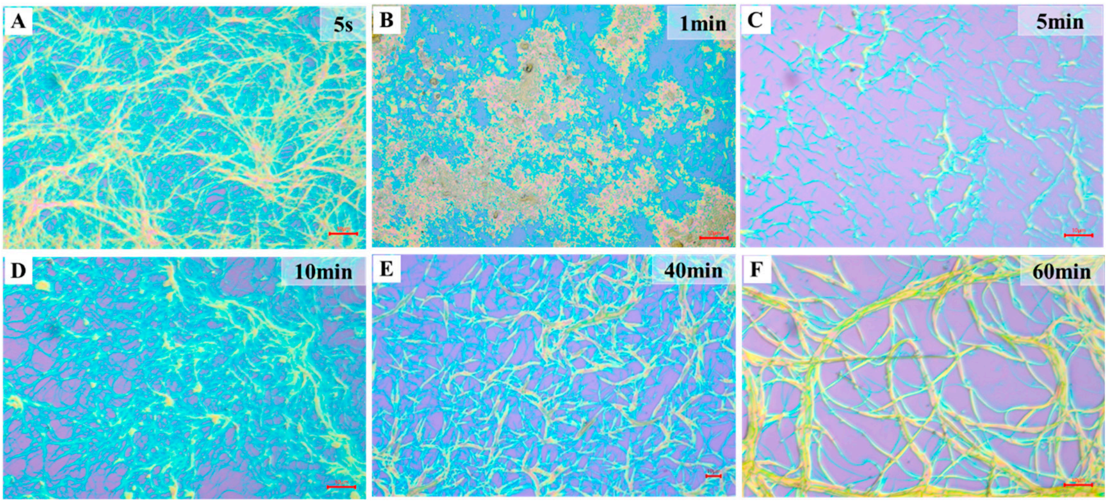

Figure S1 OM images of $CsPbBr_3$ samples prepared with different reaction times, (**A**)5 s, (**B**)1 min, (**C**)5 min, (**D**)10 min, (**E**)40 min, (**F**)60 min, Scale bar 10μm.

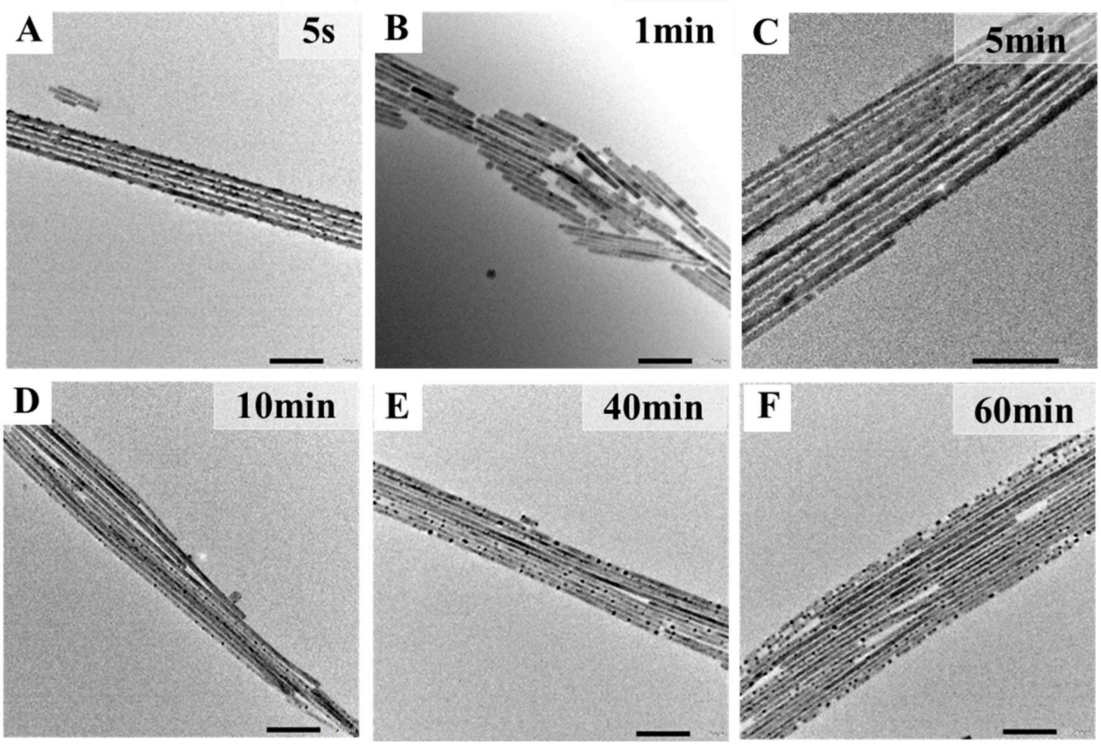

Figure S2 TEM images of $CsPbBr_3$ samples prepared with different reaction times, (**A**)5 s, (**B**)1 min, (**C**)5 min, (**D**)10 min, (**E**)40 min, (**F**)60 min, Scale bar 100nm.

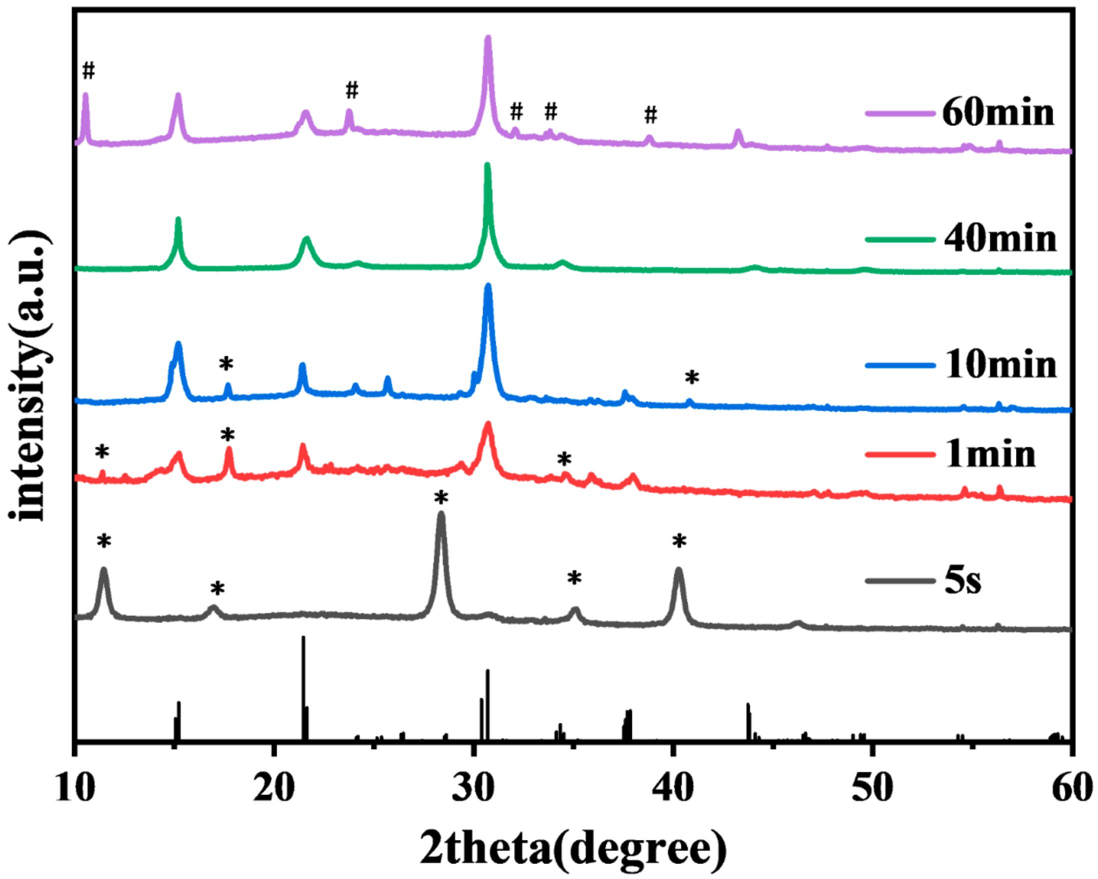

Figure S3 XRD spectra of $CsPbBr_3$ samples prepared with different reaction times. The peak labeled as "#" belongs to Cs_4PbBr_6, while the peak labeled as "*" belongs to an unknown intermediate product.

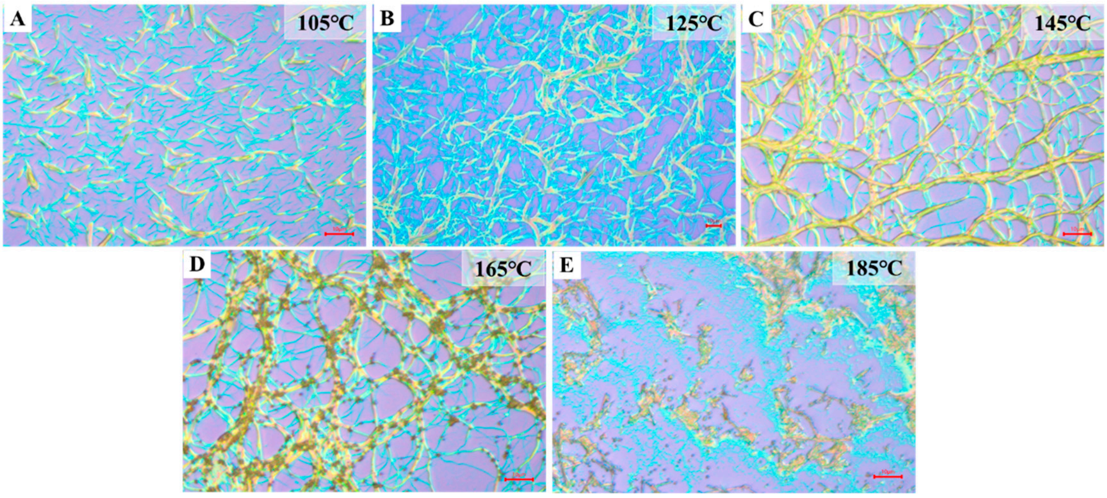

Figure S4 OM images of $CsPbBr_3$ samples prepared with different reaction temperatures, (**A**)105 °C, (**B**)125 °C, (**C**)145 °C, (**D**)165 °C, (**E**)185 °C, Scale bar 10μm.

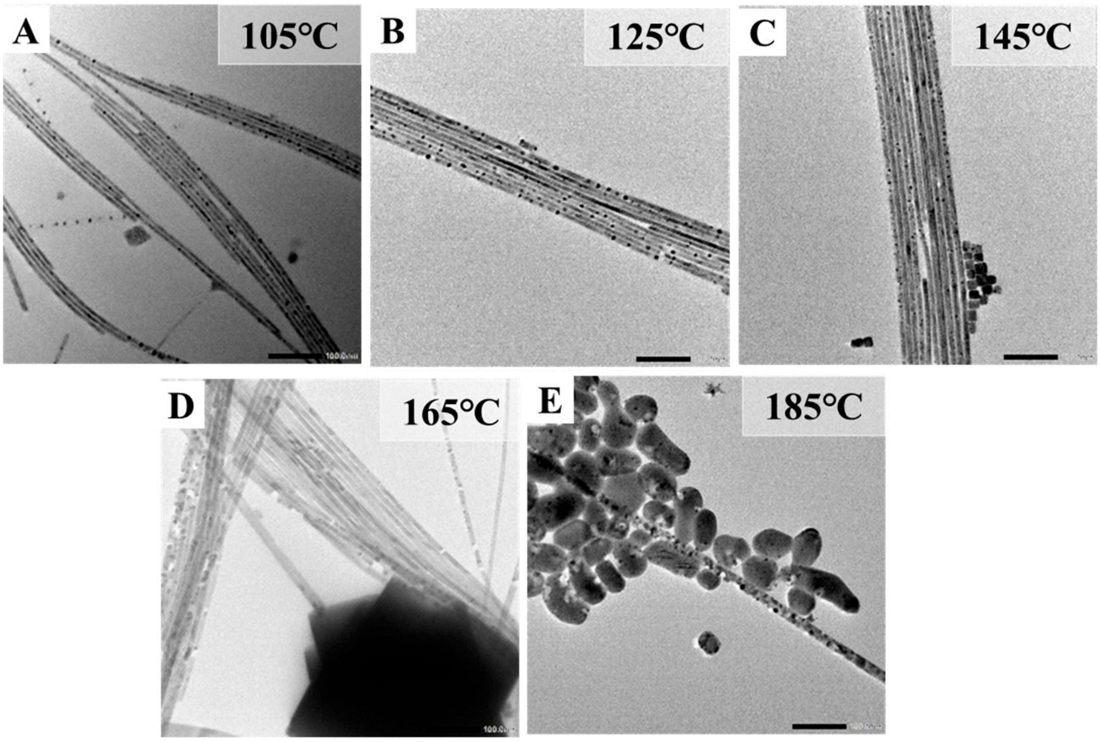

Figure S5 TEM images of $CsPbBr_3$ samples prepared with different reaction temperatures, (**A**)105 °C, (**B**)125 °C, (**C**)145 °C, (**D**)165 °C, (**E**)185 °C, Scale bar 100nm.

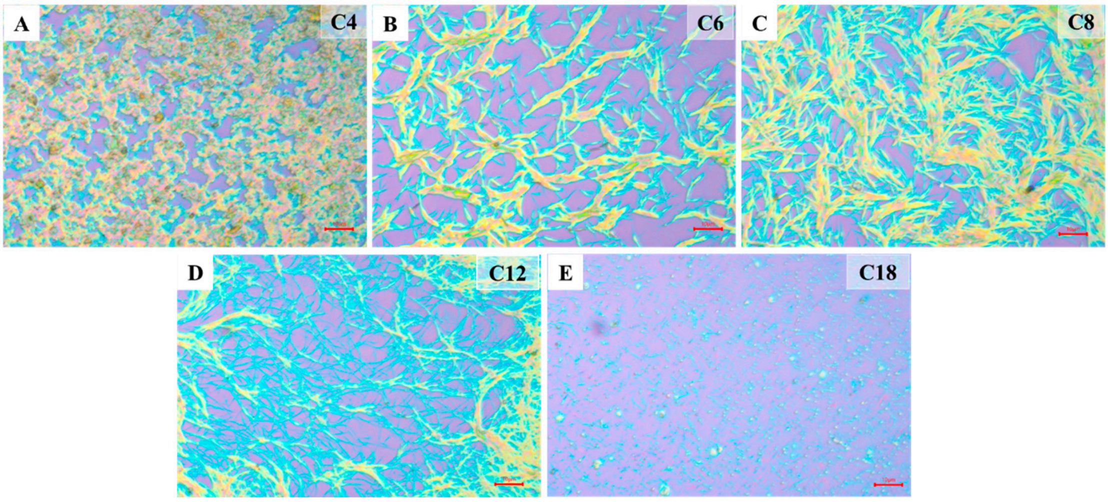

Figure S6 OM images of $CsPbBr_3$ samples prepared with organic amine ligands with different chain lengths, (**A**)C4, (**B**)C6, (**C**)C8, (**D**)C12, (**E**)C18, Scale bar 10μm.

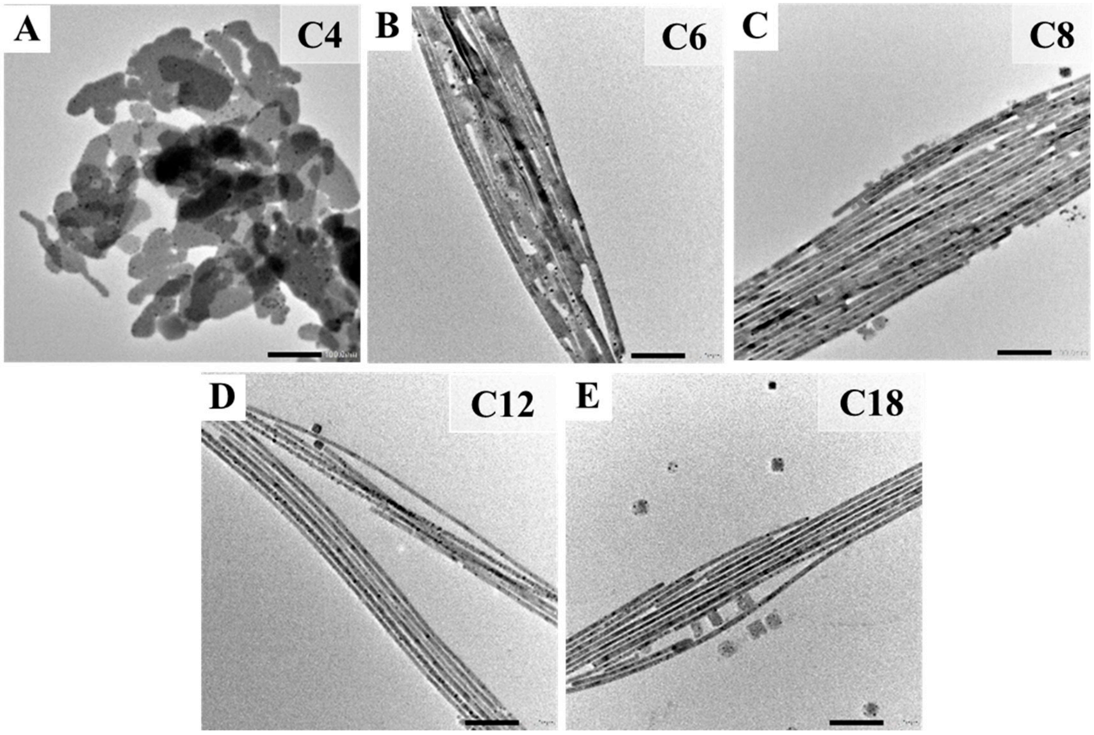

Figure S7 TEM images of $CsPbBr_3$ samples prepared with organic amine ligands with different chain lengths, (**A**)C4, (**B**)C6, (**C**)C8, (**D**)C12, (**E**)C18, Scale bar 100nm.

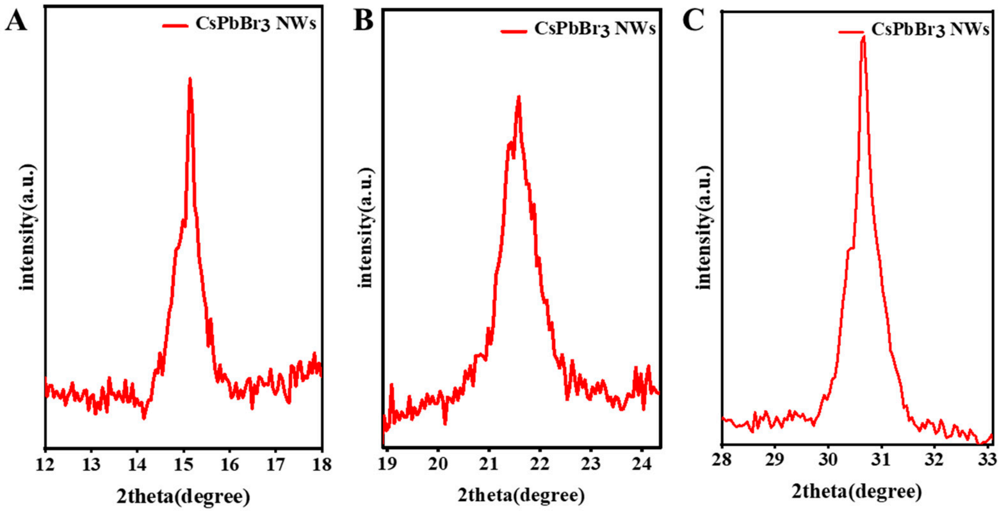

Figure S8 The locally amplified XRD spectra of the $CsPbBr_3$ NWs at (**A**)12-18°, (**B**)19-24° and (**C**)28-33°.

Table S1 EIS fitting parameters of $CsPbBr_3$ NWs

Samples	Rs (Ω cm^2)	Rc (Ω cm^2)	C_{PE} (F/cm^2)
$CsPbBr_3$ NWs	22.18	4205	$1.8212*10^{-5}$

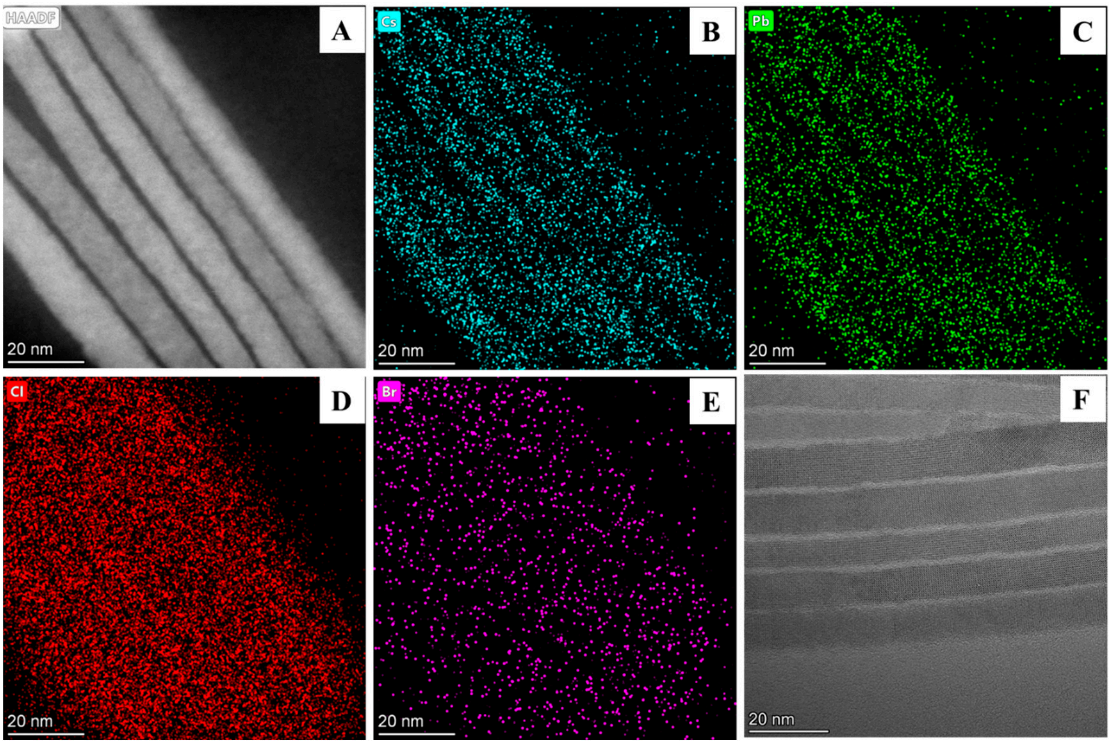

Figure S9 (**A**) HAADF-STEM and (**B-E**) STEM-EDS mapping of Cs, Pb, Cl, and Br of the $CsPbBr_xCl_{3-x}$ ($0 < x < 3$) NWs; (**F**) HRTEM image of the $CsPbBr_xCl_{3-x}$ ($0 < x < 3$) NWs.

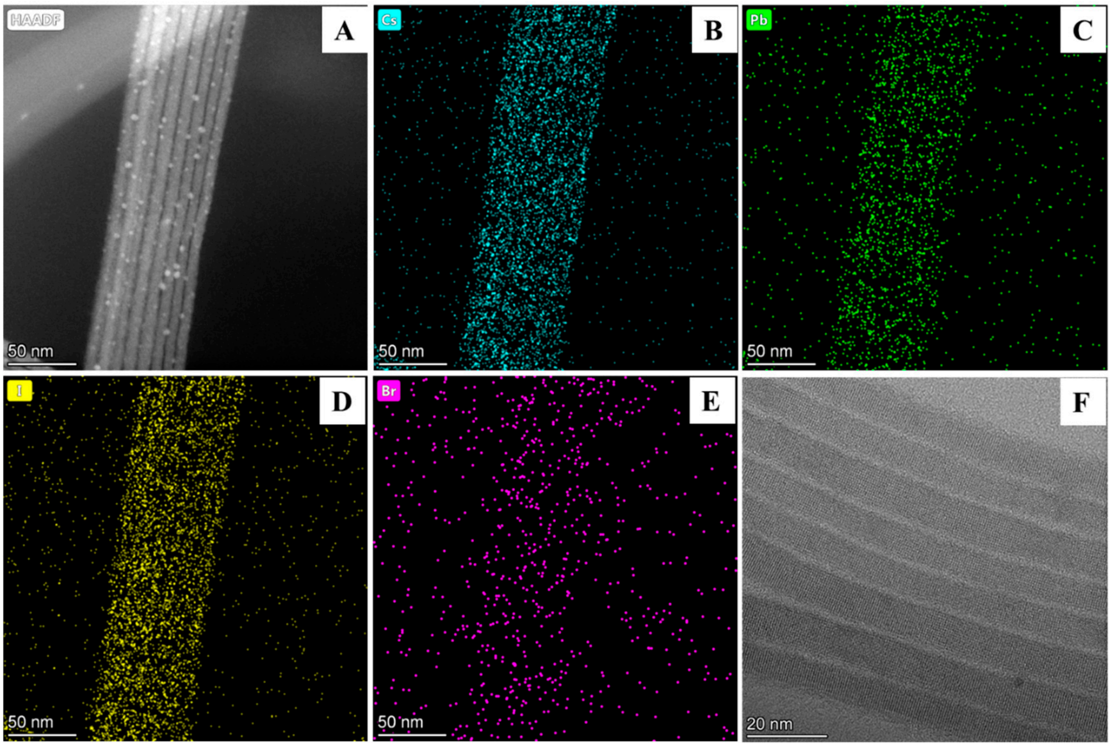

Figure S10 (**A**) HAADF-STEM and (**B-E**) STEM-EDS mapping of Cs, Pb, I, and Br of the $CsPbBr_yI_{3-y}$ ($0 < y < 3$) NWs; (**F**) HRTEM image of the $CsPbBr_yI_{3-y}$ ($0 < y < 3$) NWs.
